# Supplementary material for: A curriculum learning approach to training antibody language models
Source: bioRxiv. 2025 Mar 2:2025.02.27.640641. Preprint. [Version 1] doi: 10.1101/2025.02.27.640641 (PMC11888446; doi:10.1101/2025.02.27.640641)
Supplement: 1 [file NIHPP2025.02.27.640641V1-supplement-1.pdf]

bioRxiv preprint doi: https://doi.org/10.1101/2025.02.27.640641; this version posted March 2, 2025. The copyright holder for this preprint (which was not certified by peer review) is the author/funder, who has granted bioRxiv a license to display the preprint in perpetuity. It is made available under aCC-BY 4.0 International license.

SUPPLEMENTARY INFORMATION

	Mixed Models				***Paired Models***		***Unpaired Models***	
	Paired Data		Unpaired Data					
**Separator**	**CE Loss**	**Accuracy**	**CE Loss**	**Accuracy**	**CE Loss**	**Accuracy**	**CE Loss**	**Accuracy**
None	0.1835	**0.9510**	0.3571	**0.9133**	0.1856	**0.9507**	0.3404	0.9177
<cls>	**0.1828**	0.9509	**0.3561**	**0.9133**	**0.1851**	0.9506	0.3389	0.9179
<sep>	0.1831	0.9509	0.3565	0.9131	0.1852	0.9506	**0.3386**	**0.9180**
<cls><cls>	0.1835	0.9508	0.3601	0.9125	0.1854	0.9506	0.3423	0.9175
<sep><sep>	0.1833	0.9508	0.3604	0.9124	0.1856	0.9505	0.3427	0.9174

Table S1. CE Loss and accuracy for separator token tests. Mixed, paired-only, and unpaired-only models were trained with 5 different separators. The separators tested were: no separator, <cls>, <cls><cls>, <sep>, and <sep><sep> where <cls> is the BOS token and <sep> is a unique separator token. Separators are placed between chains in paired sequences and unpaired sequences based on the chain (end of the heavy chains and the beginning of the light chains). Models were assessed on paired and unpaired test datasets, each containing ~10k sequences.

	**Paired**		**Unpaired**	
**Unpaired %**	**CE Loss**	**Accuracy**	**CE Loss**	**Accuracy**
37.5	**0.1831**	0.9508	0.3635	0.9111
50	**0.1831**	**0.9509**	0.3565	0.9131
62.5	0.1833	**0.9509**	0.3508	0.9148
75	0.1839	0.9508	**0.3463**	**0.9164**

Table S2. CE loss and accuracy for different unpaired percentages. Mixed models were trained with increasing percentages of unpaired data. Models were assessed on paired and unpaired test datasets, each containing ~10k sequences.

bioRxiv preprint doi: https://doi.org/10.1101/2025.02.27.640641; this version posted March 2, 2025. The copyright holder for this preprint (which was not certified by peer review) is the author/funder, who has granted bioRxiv a license to display the preprint in perpetuity. It is made available under aCC-BY 4.0 International license.

Classification	Model	Accuracy	F1	AUC	AUPR	MCC
Paired HD vs CoV	unpaired	0.6555 (± 0.0020)	0.7078 (± 0.0017)	0.7443 (± 0.0015)	0.7410 (± 0.0019)	0.3331 (± 0.0042)
	constant	0.7083 (± 0.0014)	0.7169 (± 0.0028)	0.7795 (± 0.0020)	0.7751 (± 0.0029)	0.4175 (± 0.0031)
	curriculum	0.7029 (± 0.0018)	0.7067 (± 0.0020)	0.7727 (± 0.0019)	0.7679 (± 0.0033)	0.4060 (± 0.0035)
	finetuned	<u>0.7107</u> (± 0.0010)	<u>0.7137</u> (± 0.0006)	<u>0.7830</u> (± 0.0004)	<u>0.7799</u> (± 0.0013)	<u>0.4216</u> (± 0.0019)
	paired	**0.7140** (± 0.0016)	**0.7127** (± 0.0019)	**0.7855** (± 0.0005)	**0.7813** (± 0.0014)	**0.4281** (± 0.0031)
Unpaired HD vs CoV	unpaired	0.6870 (± 0.0023)	<u>0.7047</u> (± 0.0029)	0.7563 (± 0.0016)	0.7550 (± 0.0022)	0.3767 (± 0.0049)
	constant	0.6917 (± 0.0027)	**0.7081** (± 0.0031)	0.7610 (± 0.0016)	0.7554 (± 0.0016)	0.3860 (± 0.0056)
	curriculum	0.6901 (± 0.0024)	0.7044 (± 0.0030)	0.7577 (± 0.0027)	0.7547 (± 0.0032)	0.3820 (± 0.0051)
	finetuned	<u>0.6958</u> (± 0.0027)	0.7042 (± 0.0029)	<u>0.7638</u> (± 0.0008)	<u>0.7590</u> (± 0.0013)	<u>0.3923</u> (± 0.0054)
	paired	**0.7022** (± 0.0025)	0.7026 (± 0.0029)	**0.7685** (± 0.0013)	**0.7635** (± 0.0011)	**0.4044** (± 0.0050)

Table S3. Additional classification tasks to compare mixed model training methods. Results for 'HD vs CoV' specificity classification task with paired and unpaired sequences. Metrics on classification tasks are mean and standard error, with the highest values bolded and the second highest values underlined.

Classification	Dataset	Model	Accuracy	F1	AUC	AUPR	MCC
HD vs CoV	Paired	Paired-650M	0.7450 (± 0.0010)	0.7487 (± 0.0011)	0.8242 (± 0.0008)	0.8233 (± 0.0016)	0.4901 (± 0.0020)
		CurrAb	**0.7626** (± 0.0023)	**0.7669** (± 0.0016)	**0.8452** (± 0.0015)	**0.8381** (± 0.0023)	**0.5255** (± 0.0045)
	Unpaired	Paired-650M	0.7180 (± 0.0008)	0.7281 (± 0.0001)	0.7953 (± 0.0015)	0.7964 (± 0.0014)	0.4373 (± 0.0017)
		CurrAb	**0.7218** (± 0.0014)	**0.7316** (± 0.0012)	**0.8011** (± 0.0010)	**0.7992** (± 0.0014)	**0.4448** (± 0.0027)
HD vs Flu vs CoV	Paired	Paired-650M	0.6567 (± 0.0015)	0.6571 (± 0.0014)	-	-	0.4853 (± 0.0022)
		CurrAb	**0.6796** (± 0.0051)	**0.6801** (± 0.0051)	-	-	**0.5200** (± 0.0076)
	Unpaired	Paired-650M	0.6260 (± 0.0057)	0.6266 (± 0.0058)	-	-	0.4397 (± 0.0088)
		CurrAb	**0.6401** (± 0.0052)	**0.6408** (± 0.0053)	-	-	**0.4612** (± 0.0077)
Native vs Random Pairs	-	Paired-650M	0.6373 (± 0.0017)	**0.5618** (± 0.0024)	0.6920 (± 0.0011)	0.7189 (± 0.0014)	0.2926 (± 0.0036)
		CurrAb	**0.6428** (± 0.0008)	0.5462 (± 0.0029)	**0.6985** (± 0.0017)	**0.7289** (± 0.0016)	**0.3156** (± 0.0011)

Table S4. Classification tasks on 650M-parameter paired-only and curriculum models. Results specificity classification tasks (HD vs CoV' and 'HD vs Flu vs CoV') with paired and unpaired sequences and the pair classification task. Metrics on classification tasks are mean and standard error, with the highest values bolded.

bioRxiv preprint doi: https://doi.org/10.1101/2025.02.27.640641; this version posted March 2, 2025. The copyright holder for this preprint (which was not certified by peer review) is the author/funder, who has granted bioRxiv a license to display the preprint in perpetuity. It is made available under aCC-BY 4.0 International license.

Classification	Model	Accuracy	F1	AUC	AUPR	MCC
Paired HD vs CoV	IgBERT	0.6756 (± 0.0028)	0.6733 (± 0.0040)	0.7344 (± 0.0023)	0.7045 (± 0.0036)	0.3513 (± 0.0055)
	IgT5	0.6763 (± 0.0038)	0.6822 (± 0.0042)	0.7333 (± 0.0044)	0.7144 (± 0.0059)	0.3535 (± 0.0074)
	AbLang2	0.7187 (± 0.0033)	0.7237 (± 0.0027)	0.7943 (± 0.0012)	0.7764 (± 0.0016)	0.4377 (± 0.0065)
	AntiBERTa2	0.6913 (± 0.0019)	0.6982 (± 0.0019)	0.7639 (± 0.0010)	0.7642 (± 0.0011)	0.3831 (± 0.0037)
	CurrAb	**0.7626** (± 0.0023)	**0.7669** (± 0.0016)	**0.8452** (± 0.0015)	**0.8381** (± 0.0023)	**0.5255** (± 0.0045)
Unpaired HD vs CoV	IgBERT	0.6613 (± 0.0025)	0.6559 (± 0.0043)	0.7189 (± 0.0014)	0.7015 (± 0.0029)	0.3229 (± 0.0049)
	IgT5	0.6583 (± 0.0040)	0.6861 (± 0.0037)	0.7170 (± 0.0035)	0.6886 (± 0.0034)	0.3219 (± 0.0078)
	AbLang2	0.7081 (± 0.0016)	0.7140 (± 0.0021)	0.7785 (± 0.0015)	0.7574 (± 0.0013)	0.4167 (± 0.0033)
	AntiBERTa2	0.7205 (± 0.0018)	0.7193 (± 0.0027)	0.7927 (± 0.0021)	0.7893 (± 0.0018)	0.4410 (± 0.0035)
	CurrAb	**0.7218** (± 0.0014)	**0.7316** (± 0.0012)	**0.8011** (± 0.0010)	**0.7992** (± 0.0014)	**0.4448** (± 0.0027)

Table S5. Additional classification tasks to compare large-scale models. Results for 'HD vs CoV' specificity classification task with paired and unpaired sequences. Metrics on classification tasks are mean and standard error, with the highest values bolded and the second highest values underlined.

Figure	Model	A	B	Shift	k
3A-B	max1	*1*	*1*	0.6250	20
	max0.9	*0.8*	*0.9*	0.6563	20
	max0.8	*0.6*	*0.8*	0.7085	20
	max0.7	*0.4*	*0.7*	0.8137	20
3C-D	k = 10	0.4	0.7	0.8291	*10*
	k = 15	0.4	0.7	0.8166	*15*
	k = 20	0.4	0.7	0.8137	*20*
	k = 50	0.4	0.7	0.8125	*50*
3E-F	all	0.4	0.7	0.8137	20
4	curriculum	0.4	0.7	0.8166	15
5	CurrAb				

Table S6. Unpaired probability equation values for curriculum models. Corresponding figure, model, and equation values (A, B, shift, and k) are listed for each model. Italics represent the values being tested in the given figure.
